# Supplementary material for: MetaRibo-Seq measures translation in microbiomes
Source: Nat Commun. 2020 Jun 29;11:3268. doi: 10.1038/s41467-020-17081-z (PMC7324362; doi:10.1038/s41467-020-17081-z)
Supplement: Supplementary file 10 — Supplementary Data 7 [file 41467_2020_17081_MOESM10_ESM.zip › File2/Confidence_High_Taxonomy/290938_out.krona.html]

Javascript must be enabled to view this page.

members
magnitude
magnitudeUnassigned
count
unassigned
taxon
rank

290938\_out

169

2
169
superkingdom

976
169
phylum

class
169
200643

171549
169
order

family
169
815

816
genus
169

SRS011134\_contig\_number\_19524SRS011239\_contig\_number\_4537SRS012969\_contig\_number\_37996SRS013098\_contig\_number\_10604SRS016203\_contig\_number\_8610SRS017307\_contig\_number\_25573SRS017859\_contig\_number\_3650SRS018575\_contig\_number\_5843SRS020233\_contig\_number\_23301SRS021153\_contig\_number\_3092SRS023583\_contig\_number\_12001SRS024331\_contig\_number\_6023SRS046068\_contig\_number\_352SRS049995\_contig\_number\_2757SRS050026\_contig\_number\_4382SRS050299\_contig\_number\_2339SRS053649\_contig\_number\_9338SRS075878\_contig\_number\_17931SRS076756\_contig\_number\_12139SRS077392\_contig\_number\_43913SRS077849\_contig\_number\_contig-100\_691.155538SRS1041137\_contig\_number\_22537SRS147039\_contig\_number\_3971SRS971275\_contig\_number\_32803
24

28116

SRS013638\_contig\_number\_8356SRS018427\_contig\_number\_179SRS048981\_contig\_number\_1573SRS052027\_contig\_number\_10040SRS056537\_contig\_number\_43730SRS077454\_contig\_number\_2643SRS104693\_contig\_number\_66SRS143722\_contig\_number\_7642SRS893378\_contig\_number\_13450
9
species

28111
species

SRS016018\_contig\_number\_2927
1

46506
51

SRS012273\_contig\_number\_38070SRS013382\_contig\_number\_33SRS014412\_contig\_number\_19880SRS015264\_contig\_number\_1596SRS015579\_contig\_number\_19705SRS016753\_contig\_number\_820SRS016989\_contig\_number\_2537SRS018836\_contig\_number\_13923SRS019178\_contig\_number\_1572SRS019445\_contig\_number\_24267SRS020328\_contig\_number\_23103SRS020394\_contig\_number\_8971SRS022713\_contig\_number\_253SRS043768\_contig\_number\_17988SRS045739\_contig\_number\_3325SRS045826\_contig\_number\_5630SRS046717\_contig\_number\_3183SRS048164\_contig\_number\_27485SRS049402\_contig\_number\_60SRS049446\_contig\_number\_5709SRS049773\_contig\_number\_22029SRS049900\_contig\_number\_7344SRS051031\_contig\_number\_20660SRS057480\_contig\_number\_9235SRS058723\_contig\_number\_224SRS063928\_contig\_number\_69SRS064645\_contig\_number\_2259SRS074670\_contig\_number\_198SRS075021\_contig\_number\_2746SRS075984\_contig\_number\_1365SRS077086\_contig\_number\_3261SRS077335\_contig\_number\_2679SRS098061\_contig\_number\_6040SRS098073\_contig\_number\_11292SRS1041036\_contig\_number\_242SRS1041112\_contig\_number\_1825SRS1041129\_contig\_number\_10330SRS1041144\_contig\_number\_975SRS104327\_contig\_number\_624SRS1055076\_contig\_number\_13835SRS140492\_contig\_number\_4580SRS142542\_contig\_number\_12166SRS143523\_contig\_number\_3105SRS143895\_contig\_number\_30988SRS144135\_contig\_number\_16773SRS144603\_contig\_number\_7804SRS147377\_contig\_number\_2847SRS148196\_contig\_number\_43550SRS148253\_contig\_number\_4230SRS148817\_contig\_number\_9980SRS893366\_contig\_number\_7722
species

2292913
species
1

SRS019286\_contig\_number\_5174

species
5

SRS017521\_contig\_number\_20430SRS019601\_contig\_number\_12635SRS043411\_contig\_number\_1609SRS098655\_contig\_number\_1831SRS147653\_contig\_number\_425
817

47678
3

SRS017701\_contig\_number\_2809SRS078665\_contig\_number\_2149SRS147977\_contig\_number\_1814
species

2292950
species
8

SRS015694\_contig\_number\_14569SRS017247\_contig\_number\_7996SRS024075\_contig\_number\_8844SRS062654\_contig\_number\_16090SRS098644\_contig\_number\_25136SRS143148\_contig\_number\_111SRS148319\_contig\_number\_637SRS148784\_contig\_number\_1086

1946089
species

SRS019068\_contig\_number\_52375SRS064276\_contig\_number\_41283
2

93975
species

SRS013940\_contig\_number\_3953SRS014923\_contig\_number\_5883SRS050422\_contig\_number\_15937SRS054059\_contig\_number\_874SRS075821\_contig\_number\_1016SRS1041138\_contig\_number\_71SRS893172\_contig\_number\_12165
7

2

SRS097920\_contig\_number\_3393SRS097958\_contig\_number\_12991
species
246787

1

SRS014613\_contig\_number\_3397
species
2292193

1

SRS893342\_contig\_number\_340
species
338188

species
2

SRS019496\_contig\_number\_386SRS104400\_contig\_number\_1658
821

2292281
1

SRS022609\_contig\_number\_43330
species

329854
1

SRS893358\_contig\_number\_432
species

species
1

SRS064232\_contig\_number\_301
2292920

species

SRS011302\_contig\_number\_29278SRS014313\_contig\_number\_5110SRS015190\_contig\_number\_1803SRS018817\_contig\_number\_2840SRS042628\_contig\_number\_33234SRS050752\_contig\_number\_5690SRS050801\_contig\_number\_3870SRS051882\_contig\_number\_18317SRS053214\_contig\_number\_2592SRS055017\_contig\_number\_1224SRS056259\_contig\_number\_3187SRS056273\_contig\_number\_8745SRS063518\_contig\_number\_18914SRS064973\_contig\_number\_1186SRS074586\_contig\_number\_1320SRS077730\_contig\_number\_10876SRS078242\_contig\_number\_25753SRS098881\_contig\_number\_18543SRS1041033\_contig\_number\_15173SRS1041039\_contig\_number\_20392SRS1041091\_contig\_number\_12961SRS1041133\_contig\_number\_8625SRS1041157\_contig\_number\_6342SRS104975\_contig\_number\_7094SRS1054716\_contig\_number\_11834SRS143085\_contig\_number\_372SRS144297\_contig\_number\_13855SRS144714\_contig\_number\_19973SRS146812\_contig\_number\_61640SRS147271\_contig\_number\_30429SRS147614\_contig\_number\_9142SRS148970\_contig\_number\_72SRS149075\_contig\_number\_66SRS149181\_contig\_number\_20378SRS149244\_contig\_number\_6566SRS149325\_contig\_number\_11185SRS893270\_contig\_number\_12545SRS893369\_contig\_number\_4607SRS893380\_contig\_number\_561SRS893382\_contig\_number\_5607
40
820

1

SRS1041164\_contig\_number\_560
species
371601

1263038

SRS1055083\_contig\_number\_6141
1
species

818
species

SRS015794\_contig\_number\_57SRS018351\_contig\_number\_9289SRS104636\_contig\_number\_15532
3

species

SRS1041134\_contig\_number\_13048
1
674529

1638780
3

SRS014948\_contig\_number\_20432SRS050925\_contig\_number\_14089SRS148091\_contig\_number\_9962
species
